# Deep Denoising of Raw Biomedical Knowledge Graph From COVID-19 Literature, LitCovid, and Pubtator: Framework Development and Validation

**DOI:** 10.2196/38584

**Published:** 2022-07-06

**Authors:** Chao Jiang, Victoria Ngo, Richard Chapman, Yue Yu, Hongfang Liu, Guoqian Jiang, Nansu Zong

**Affiliations:** 1 Department of Computer Science and Software Engineering Auburn University Auburn, AL United States; 2 Center for Innovation to Implementation VA Palo Alto Health Care System Sacramento, CA United States; 3 Stanford Health Policy Stanford School of Medicine Stanford University Stanford, CA United States; 4 Freeman Spogli Institute for International Studies Stanford University Stanford, CA United States; 5 Department of Artificial Intelligence and Informatics Research Mayo Clinic Rochester, MN United States

**Keywords:** adversarial generative network, knowledge graph, deep denoising, machine learning, COVID-19, biomedical, neural network, network model, training data

## Abstract

**Background:**

Multiple types of biomedical associations of knowledge graphs, including COVID-19–related ones, are constructed based on co-occurring biomedical entities retrieved from recent literature. However, the applications derived from these raw graphs (eg, association predictions among genes, drugs, and diseases) have a high probability of false-positive predictions as co-occurrences in the literature do not always mean there is a true biomedical association between two entities.

**Objective:**

Data quality plays an important role in training deep neural network models; however, most of the current work in this area has been focused on improving a model’s performance with the assumption that the preprocessed data are clean. Here, we studied how to remove noise from raw knowledge graphs with limited labeled information.

**Methods:**

The proposed framework used generative-based deep neural networks to generate a graph that can distinguish the unknown associations in the raw training graph. Two generative adversarial network models, NetGAN and Cross-Entropy Low-rank Logits (CELL), were adopted for the edge classification (ie, link prediction), leveraging unlabeled link information based on a real knowledge graph built from LitCovid and Pubtator.

**Results:**

The performance of link prediction, especially in the extreme case of training data versus test data at a ratio of 1:9, demonstrated that the proposed method still achieved favorable results (area under the receiver operating characteristic curve >0.8 for the synthetic data set and 0.7 for the real data set), despite the limited amount of testing data available.

**Conclusions:**

Our preliminary findings showed the proposed framework achieved promising results for removing noise during data preprocessing of the biomedical knowledge graph, potentially improving the performance of downstream applications by providing cleaner data.

## Introduction

The effects of the COVID-19 pandemic linger in 2022—it affected over 11.6 million people globally in the past year, and accounted for >2.5 million deaths in more than 220 countries [1]. With the continuous accumulation of peer-reviewed publications on the topic, a literature hub serves as a means to track the most up-to-date scientific information about the virus [2]—encompassing research on the treatment, diagnosis, and prevention of COVID-19. A knowledge base built upon the integration of biomedical entities from such a literature hub would provide tremendous value in the exploration of explicit or implicit associations among diverse biomedical entities as investigators attempt to answer clinical questions related to COVID-19. A number of recently published journal articles have included graph-based analysis of COVID-19 data sets [3]. For example, Groza [4] analyzed how a semantically annotated data set would be helpful in detecting and preventing potentially harmful misinformation regarding the spread of COVID-19 based on CORD-19-on-FHIR (a linked data version of the COVID-19 Open Research Dataset [CORD-19] data represented in FHIR RDF by mining the CORD-19 data set and adding semantic annotations) [5].

Most knowledge graphs constructed for COVID-19 are currently based on the co-occurring biomedical entities reported in recent literature. A knowledge graph of co-occurring concepts, such as the one created by Oniani et al [6], can help researchers find associations among genes, drugs, and diseases related to COVID-19. Using knowledge graphs with heterogeneous biomedical associations (eg, gene-drug, disease-drug, drug–side effect) in these types of applications, however, results in a high probability of false-positive predictions because co-occurrence in literature does not always mean there is a true biomedical association between the two entities. These co-occurrence edges are therefore considered “noise” due to their untrue associations. For example, the term “glucose” may co-occur with the term “yellow fever,” but there is no real medical association between the two terms. Noise removal can be beneficial for downstream applications, such as link prediction [7], representation learning [8,9], and node classification [10].

The manual processes of cleaning data and removing noise are resource intensive. Therefore, an automated denoising method is ideal in facilitating the curation of knowledge graphs. Existing methods for denoising knowledge graphs can be divided into two groups: internal and external [11]. For the internal method, the predefined semantics or rules [12] are used for nonnumerical data. Outlier detection [13] removes noise by modeling true data as a distribution for numerical data. As for external methods, a pretrained graph neural network integrates heterogeneous data sources [14] to not only improve the performance of link prediction but also reduce the training time of the existing graph neural network model. In this paper, our methodology can be categorized as an internal method where data augmentation with a generative adversarial network (GAN) removes noise. GAN has been widely applied in medical imaging process [15] to denoise computed tomography images based on GAN with Wasserstein distance and perceptual similarity. Zhou et al [16] previously showed improvement of ultrasonic image quality and noise reduction caused device limitations through the construction of a two-stage GAN. Other than the application of generating images, GAN has mainly been used for generating discrete medical data to contribute to the scenario of diagnosis of a disease with few labels [17] or unbalanced classification [18]. To the best knowledge of the authors, our study is the first study that uses GAN to denoise a biomedical knowledge graph.

Here, we propose a framework that generates a similar graph from a raw knowledge graph to distinguish the true and false edges of association based on generative-based deep neural networks. Two recent generative-based models, Cross-Entropy Low-rank Logits (CELL) [19] and a generative-based graph method (NetGAN) [20], have been adopted as a component to remove noise and retain true associations within two data sets: (1) a synthetic data set generated from CORA-ML [21] with the same preprocessing as in NetGAN [20]; and (2) a real data set constructed from CORD-19-on-FHIR data sets with heterogeneous biomedical associations (ie, chemical-disease, gene-disease, gene-chemical associations) [5]. Our study shows the proposed method achieved promising results in the classification tasks for separating the true and negative edges.

## Methods

### Problem Definition

Given a network G(V, E), where *V* stands for a set of vertices (ie, biomedical concepts in the literature) and *E* represents the edges among two vertices (ie, the co-occurrence of two concepts), two kinds of edges exist, which are denoted as *L* (known true associations) and *U* (unknown true associations). We note that if no edge exists between two vertices, this will be considered a false association. The aim is to find the true associations among *U* (ie, denoise *U*). Specifically, a proposed method should have the capacity to determine whether unknown true associations from *U* are true associations or false.

### Framework

#### Overview

As this problem could be considered a classification of an unknown edge with a small number of known true associations and a large number of unknown true associations, we defined this classification problem as few-shot learning [22]. We proposed a framework that used generative-based deep neural networks (eg, NetGAN and CELL) to denoise the unknown true associations in *U* based on similar networks generated. This framework was divided into 3 parts. We first briefly describe the GAN-based denoising graph adopted following the development of the framework, followed by an introduction of data preparation, which involved two strategies: (1) synthetic data generation and (2) real data set collection and annotation. A comprehensive design of our experiments was then conducted to verify our assumptions.

#### Denoising Based on Generative-Based Deep Neural Networks

We adopted NetGAN to generate a new network that would be used to distinguish the unknown associations in the raw training data (ie, graph). To achieve this, we randomly sampled walks from the raw graph consisting of unlabeled edges and trained a generator to learn the walks sampled and a discriminator on how to separate a real walk from a fake one. After achieving equilibrium among the discriminator and generator, the random walk sample from the generator was used for filtering the unreal edge in the raw graph. As determined in previous work by other researchers [19], sampling enough random walks was sufficient to reconstruct the graph. Both the generator and discriminator used the long short-term memory (LSTM) architecture [23] and were trained with the Wasserstein loss [24]. The generator *G* generated large numbers of random walks (node sequence) of fixed length. The discriminator *D* distinguished the sequence of the nodes sampled from *G* and *x* that were sampled from the real graph (including unlabeled associations) with randomly started nodes. *D* and *G* played the following minimax game with the value function *V(D, G)*:



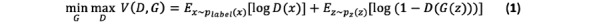



Finally, the *D* generated a similar authentic graph network that could not be distinguished by the discriminator *G*.

To generate the probability of the edges, CELL approximated it with a score matrix *S*, which was computed by 
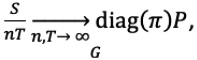
 where *n* is the number of random walks, *T* is each length of a random walk, and diag(π) is the stationary distribution matrix. *P* is a transition matrix that approximates the unbiased random walk used in NetGAN. *P* can be low-rank approximated by *W*, which is the logit transition matrix and is solved by the objective function as:



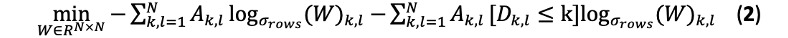



where *A* is the adjacency matrix and s.t. rank(W) ≤ *H*. In practice, we further adapted node2vec [25] for the random sampling process in NetGAN and constrained the edge generation length with *k* in the above loss function in CELL.

#### Data Preparation

##### Overview

**Figure 1:**
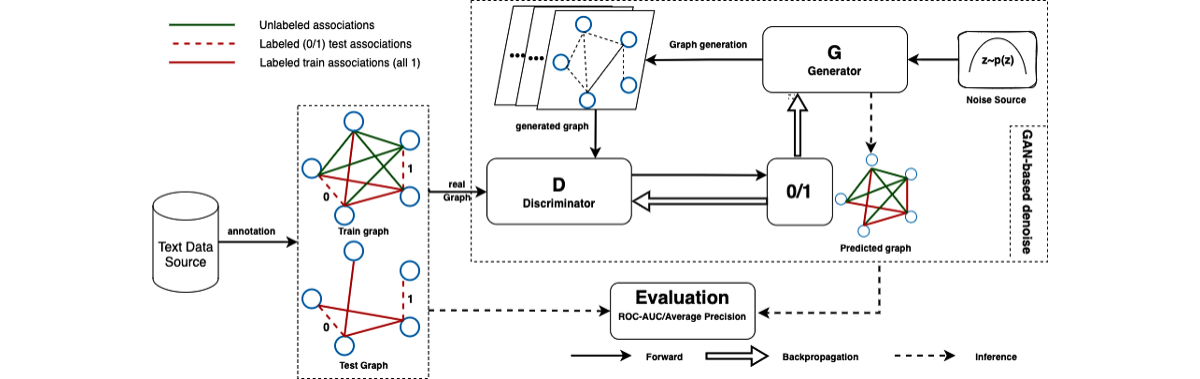
Overview of our investigation process. GAN: generative adversarial network; ROC-AUC: area under the receiver operating characteristic curve.

**Figure 2:**
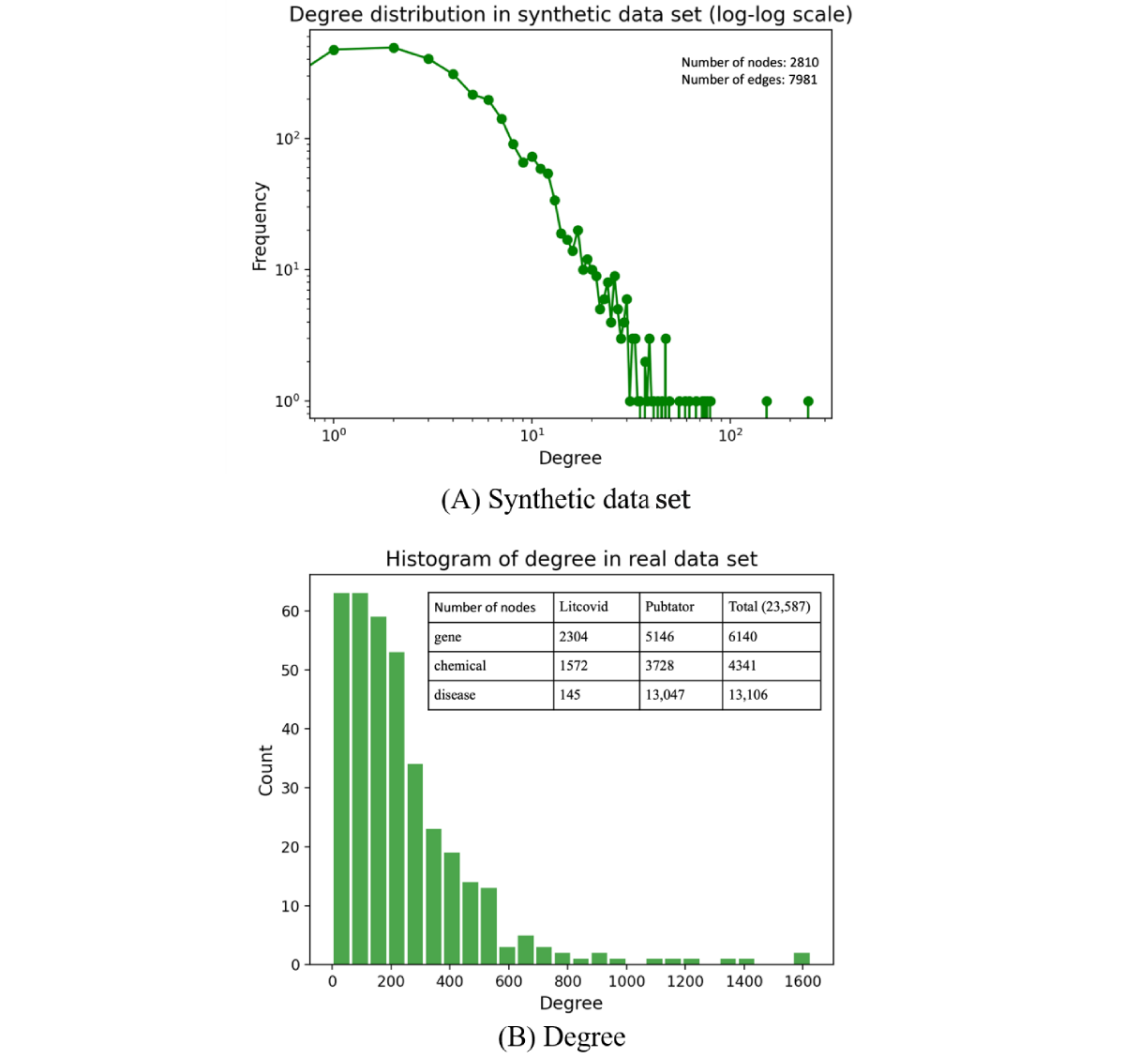
Histogram of degree distribution in the synthetic data set and real data set.

We generated two data sets for this study: (1) a synthetic data set based on CORA-ML, and (2) a real data set extracted from CORD-19-on-FHIR data sets [5]. First, we defined two types of associations: labeled associations denoted as (*L*) (red colored) and unlabeled associations represented as (*U*) (green colored) (Figure 1), based on two types of association. This was used to construct our training and test graphs. The training graph consisted of both the labeled (*L*) and unlabeled (*U*) associations, while there were only labeled (*L*) associations in the test graph, as we need the ground truth for evaluating the performance of our proposed methods. The histogram of each data set is given below, where Figure 2A is the synthetic data set. This does not include the false associations added in our subsequent experiments. Figure 2B shows the histogram of degree distribution in the real data set.

##### Synthetic Data Set

The synthetic data set was generated based on CORA-ML with the same preprocessing work as NetGAN [20]; we chose the largest connected component in the graph. The final total number of nodes and edges is shown in the top right corner of Figure 2A. To test our proposed methods, we took as ground truth the existing edges as true associations and the nonexistence edges as false associations. Detailed synthetic data processes can be found in Figure S1 in Multimedia Appendix 1.

##### Real Data Set

From CORD-19-on-FHIR [5], we used two annotated networks (LitCovid [26], Pubtator [27]), extracting the COVID-19–related terms in our SPARQL query with 3 types of biomedical concepts (ie, gene, chemical, mutation/disease). After merging identical IDs from both LitCovid and Pubtator, we were able to obtain a new data set with a total of 23,578 nodes (Figure 2B). Finally, we randomly chose a proportional number of edges with a total of 500 associations (ie, chemical-disease, gene-disease, gene-chemical associations) from a total of 288,270 edges in the whole graph and manually labeled them as our labeled data set. Detailed data preprocessing and degree distribution for each type of association can be found in Supplementary 2 in Multimedia Appendix 1.

#### Experiment Design

##### Overview

We conducted experiments on both a synthetic data set (ie, CORA-ML) and a real data set extracted from CORD-19-on-FHIR to investigate the capability of our proposed methods of incorporating unlabeled information for improving the link prediction performance despite limited annotation. We analyzed the performance of our models with multiple tasks based on two types of ratios to mimic the percentages of noise and annotation during the data curation: (1) noise ratio (NR), the percentage of true and false associations in the unlabeled edges; and (2) annotation ratio (AR), the percentage of training and testing associations in the labeled edges.

##### Task 1: Test of AR Over the Synthetic Data Set

We wanted to understand how many annotations were needed during the data curation for our proposed method to predict the true and false associations. We set a fixed NR and evaluated the performance of the tested method in two cases: one included the unlabeled data (ie, training set = labeled true and false associations + unlabeled associations), and the other did not include the unlabeled data (ie, training set = labeled true and false associations). The unlabeled associations were taken as true associations for training. In the experiment, we tested the performances based on different AR to mimic the percentage of the annotations already completed during the data curation. In practice, the AR varied from 1:9 to 9:1. For each ratio, we repeated the test 10 times with a random sampling of the training and testing sets to get the average results.

##### Task 2: Test of NR Over the Synthetic Data Set

In this task, we wanted to understand how many false associations were deemed as true associations in unlabeled data for training because it affected the prediction performance of the proposed method. We wanted to see whether the proposed method was robust enough to learn useful information for prediction, especially from unlabeled edges with more noise. With a fixed AR of 1:1, we tested the proposed method when there were more false edges than real edges in the unlabeled data. In practice, the NR varied from 1:1 to 1:9.

##### Task 3: Test Over the Real Data Set

After the same training of annotation, two of the authors (CJ and YY) manually labeled 500 of the 288,270 edges to simulate an extreme use case for data curation, and another author (VN) verified the annotation by random sampling the edges. Among the 500 edges, the 3 types consisted of chemical-disease, gene-chemical, and gene-disease. Each edge was marked as true, false, and unknown. In practice, the annotations for gene-chemical were excluded and marked as unknown in the final evaluation after the authors had a discussion and reached a consensus that those annotations were conducted without enough confidence. Thus, in our final result report of the receiver operating characteristic curve, we only considered 2 types of associations: chemical-disease and gene-disease.

#### Setting and Evaluation Metrics

For each proposed method (ie, NetGAN and CELL), a grid search strategy was adapted for obtaining the best hyperparameters. In our experiment, we defined the search range by referencing the original settings in the articles. For NetGAN, the parameter ranges for the grid search are specified as walk *p* = {0.01, 0.1, 1, 10, 100} and *q* = {0.01, 0.1, 1, 10, 100}. For CELL, the parameter ranges are specified as rank *H* = {9, 20}, learning rate *lr* = {0.01, 0.05, 0.1}, and weight decay *weight_decay_* = {1*e* – 5, 1*e* – 6, 1*e* – 7}. In practice, the origin NetGAN was obtained from [28], and the origin CELL was obtained from [29].

In the evaluation step, we chose the area under the receiver operating characteristic curve (AUC ROC) and average precision (AP) as the metrics of link prediction for our proposed methods in both synthetic and real data sets. In the implementation, both AUC ROC and AP scores were calculated by scikit-learn [30]. The visualization of predicted results in our real data set was a plot made with Cytoscape [31], an open-source software platform for visualizing complex networks and integrating these with any type of attribute data.

## Results

### Task Evaluation Outcomes

#### Task 1: Comparison of the Link Prediction Results in a Graph With/Without the Unlabeled Associations Among Different ARs

**Figure 3:**
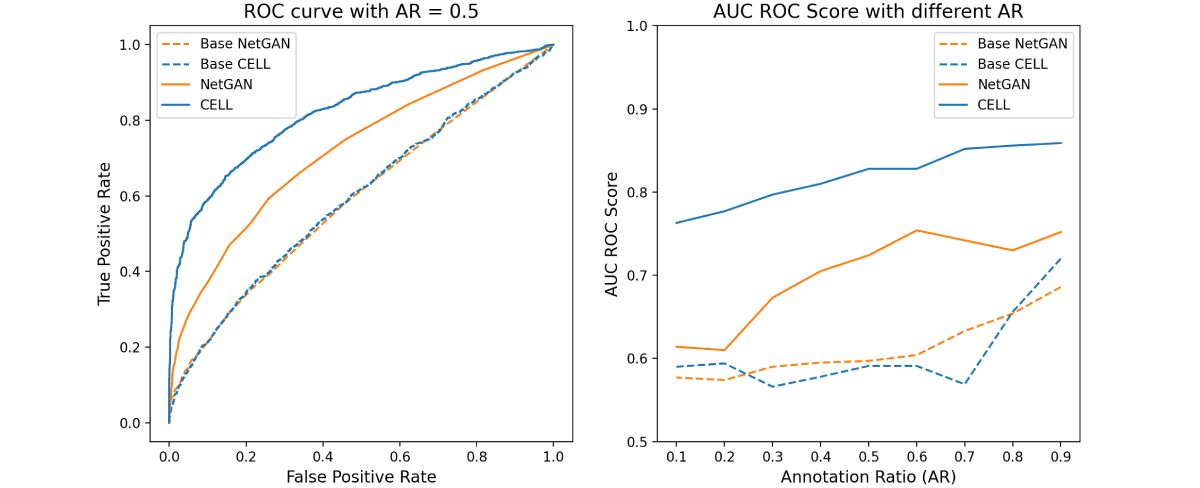
AUC ROC performance of NetGAN and CELL with/without unlabeled information. AR: annotation ratio; AUC ROC: area under the receiver operating characteristic curve; CELL: Cross-Entropy Low-rank Logits.

We conducted our experiments in two scenarios. One was the base case (dashed line in Figure 3) where we tested our models without using the unlabeled information, without explicitly stating it as the base case; all of our statements in the following section would be the default case (solid line in Figure 3) that indicated that we included the unlabeled associations in the link prediction tasks. We reported the AUC ROC score in Figure 3. Here, the left subfigure displayed the AUC ROC curve with a fixed AR of 0.5. The dashed line named “Base NetGAN” indicates the method of NetGAN that did not incorporate the unlabeled information. “Base CELL” is the method that CELL runs in the base case. There was little difference between the two methods when considering the base case with an AUC ROC score of 0.597 for NetGAN and 0.591 for CELL. However, when unlabeled information was taken into consideration, both methods achieved better performance compared to the base case (the AUC ROC score of NetGAN was 0.724, while CELL achieved a score of 0.828). The right side of Figure 3 shows the performance of the proposed methods in different ARs ranging from 0.1-0.9. We determined that CELL had overall better performance.

#### Task 2: How Do the Models Perform With Different NRs in the Unlabeled Edges?

**Figure 4:**
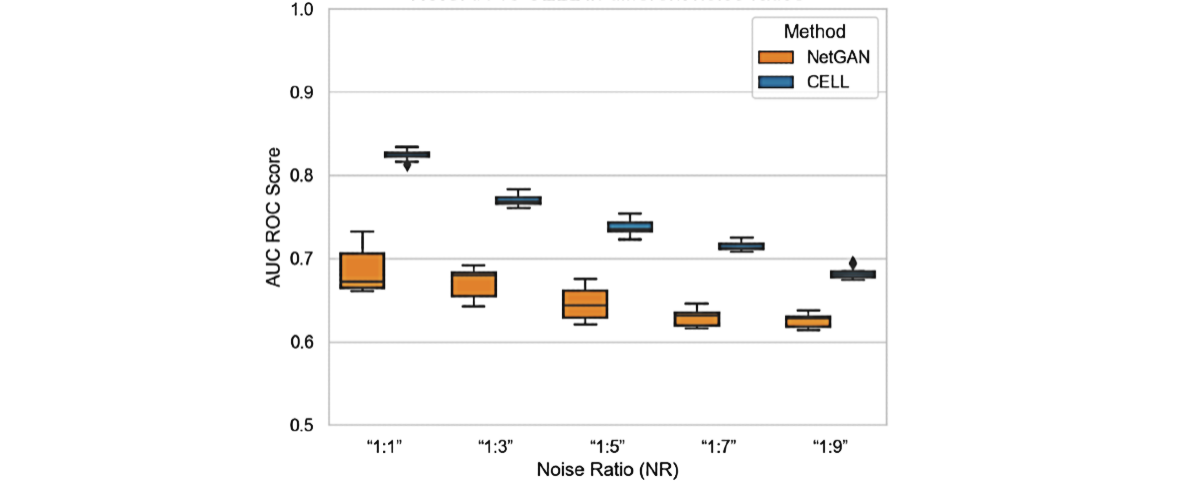
Performance in terms of AUC score at different noise ratios. AUC: area under the curve; AUC ROC: area under the receiver operating characteristic curve; CELL: Cross-Entropy Low-rank Logits.

We tested the performance of methods in which the unlabeled information contained a different ratio of noise 10 times (Figure 4). CELL demonstrated exceptional performance when the NR was 1:1. Even in the extreme case where the true versus false ratio reached 1:9, CELL still had better performance compared to NetGAN with an area under the curve (AUC) score of around 0.7. CELL had less variance in performance compared with NetGAN at all NRs. In other words, CELL had a relatively better capability and stability to use unlabeled data compared with NetGAN when dealing with the complexity of the NR in unknown information.

#### Task 3: The Performance of Proposed Models in Our Collected Real Data Set

**Figure 5:**
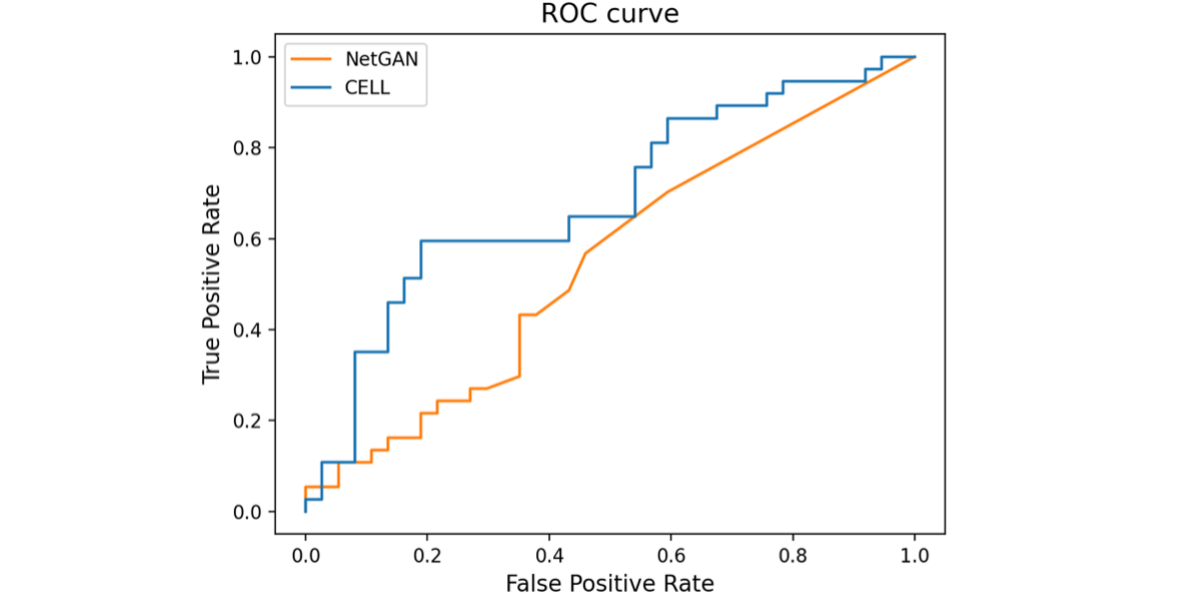
Performance on real data set. ROC: receiver operating characteristic curve; CELL: Cross-Entropy Low-rank Logits.

**Figure 6:**
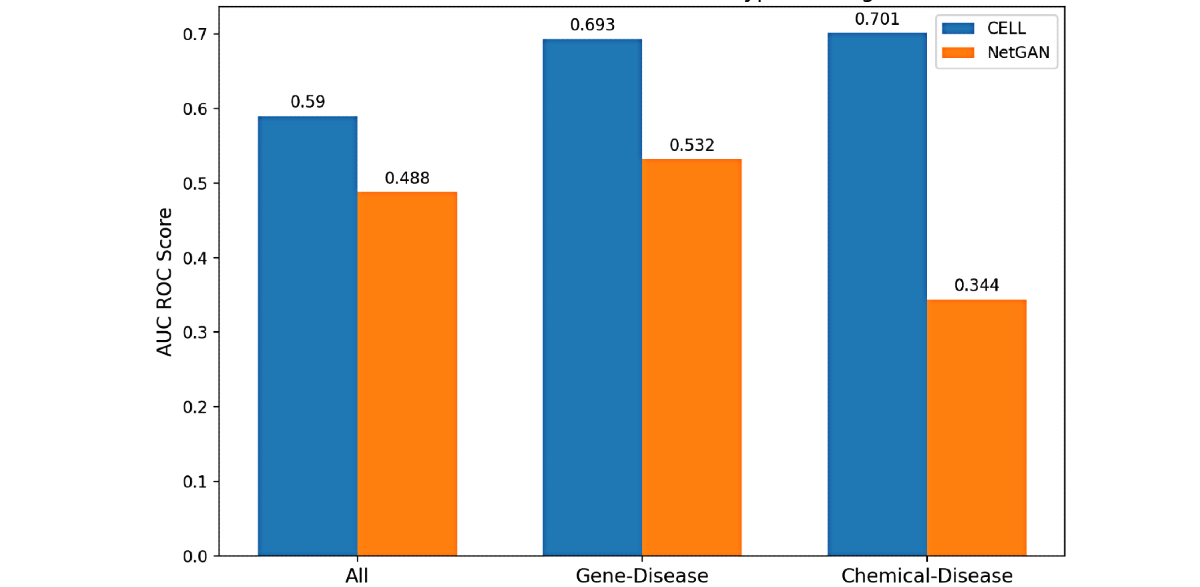
AUC ROC (area under the receiver operating characteristic curve) score for different types of associations in the real data set. CELL: Cross-Entropy Low-rank Logits.

After our exploration of our methods in task 2, we conducted our methods on a real data set. Although the NR was unknown in our real data set, the proposed methods still performed better than random classification with the incorporation of unknown associations. In addition, compared with NetGAN, CELL still had an impressive result with an AUC ROC of up to 0.706 when the test and train ratio was 1:1 and the unknown association occupied about 99.95% as shown in Figure 5. The good performance of CELL showed that it had an excellent capability to predict the true association with the use of unlabeled data. We reported the AUC ROC value of each type of association separately in Figure 6. Combining Figure S2 in Multimedia Appendix 1 of edge degree of each type of association, we concluded that, as the degree is larger, there would be more noise contained in each edge. Thus, the results would be affected correspondingly. The average precision performance for our proposed models in our synthetic and real data sets can be found in Supplementary 3 in Multimedia Appendix 1.

#### Denoised Knowledge Graph Generated From the Real Data Set

**Figure 7:**
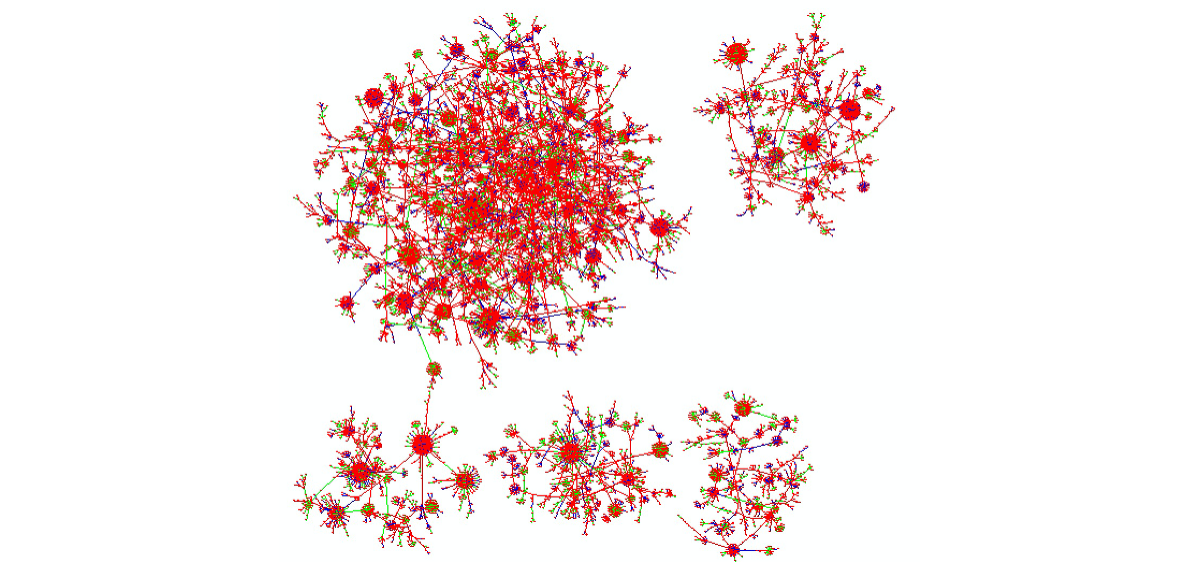
Visualization of the predicted knowledge graph based on the real COVID-19–related data set.

We trained the adapted NetGAN with the whole real data set, and plotted the predicted denoised knowledge graph in Figure 7, where the edges are generated based on the score matrix calculated following the generation method used in NetGAN [12]. There are a total of 21,016 edges in our visualization consisting of gene-chemical (7562), gene-disease (7613), and chemical-disease (5841). Three different colors (red, green, and blue) stand for three different types of associations/edges (gene-chemical, gene-disease, chem-disease). The source file for the prediction can be found at [32].

## Discussion

### Principal Results

In this study, we proposed a method to automate the denoising of a knowledge graph generated via the counting of co-occurrence from biomedical literature. Our work can be considered as the preprocessing part for the curation of the knowledge graph. We adopted state-of-the-art generative-based graph methods, NetGAN and CELL, to leverage the unlabeled co-occurring biomedical entities in the training process by the perturbation of the original graph in the determination of an unknown edge. Two data sets (ie, synthetic and real data sets) were used to evaluate proposed methods in 3 link prediction tasks, and our experiments achieved promising results with both synthetic and real data sets.

### Limitations

Despite the capability and stability of the methods used in this study, there are a few limitations that need to be discussed.

First, the associations labeled in the real data set are limited due to limited resources. In addition, due to the reality of vagueness or missing concepts in the biomedical literature, there will be some bias. A large sample of annotated associations may provide a solution to reduce this bias and thus is needed for our future work. One way to potentially accomplish this goal would be to use natural language processing methods to standardize the concepts prior to annotation, which may improve the construction of knowledge graph input to our methods. Another way includes collaborating with professional annotators to both increase the number of annotations as well as improve the quality.

Second, while we have achieved notable improvement with AUC around 0.7 in our real data set compared with random classification, there is still a gap between the experimental results in a controlled environment compared to the adaptation of the proposed methods for data curation in real-world scenarios. Performance improvement is still needed. The complexity of our investigated algorithm comes from the module of LSTM, which generates random walks for reconstructing the graph. An adaptation of binary neural networks [33] that directly produces the discrete adjacency matrix for the graph may have the potential to significantly improve the efficacy of our investigated methods as reconstruction of the adjacency matrix from random walks will not be needed. Another potential direction for improving the performance of removing noise in our investigated methods could be looking into the possibilities of transfer learning or external methods as we discussed previously, such as in [34]. By importing prior knowledge into the process of graph generation, we could employ the knowledge from an already built data set [35] to help us remove the false associations when constructing our biomedical graph.

Third, our evaluation was based on the logic of classifying the true or false associations directly, and was intentionally not focused on the impact evaluation of the denoised data sets generated in our work on downstream applications (eg, prediction for drug-target association and protein-protein interaction). Although we assume the performance will be improved in those applications [36], we acknowledge that there has not yet been any scientific proof to support that. The whole data stream, including the methods of data processing, data curation (ie, denoising method proposed in this study), and application, needs to be investigated further to fill this gap, which could provide convincing evidence of the impact of our proposed method for denoising knowledge base construction.

## Appendix

Multimedia Appendix 1 Supplementary materials.

## Abbreviations

AP: average precision
AR: annotation ratio
AUC: area under the curve
AUC ROC: area under the receiver operating characteristic curve
CELL: Cross-Entropy Low-rank Logits
CORD-19: COVID-19 Open Research Dataset
GAN: generative adversarial network
LSTM: long short-term memory

## References

[1] Coronavirus disease (COVID-19). World Health Organization.

[2] Chen Q, Allot A, Lu Z (2020). Keep up with the latest coronavirus research. Nature.

[3] El Mouden ZA, Taj RM, Jakimi A, Hajar M (2020). Towards using graph analytics for tracking covid-19. Procedia Comput Sci.

[4] Groza A Detecting fake news for the new coronavirus by reasoning on the Covid-19 ontology. ArXiv.

[5] CORD-19-on-FHIR. GitHub.

[6] Oniani D, Jiang G, Liu H, Shen F (2020). Constructing co-occurrence network embeddings to assist association extraction for COVID-19 and other coronavirus infectious diseases. J Am Med Inform Assoc.

[7] Zong N, Wong RSN, Yu Y, Wen A, Huang M, Li N (2021). Drug-target prediction utilizing heterogeneous bio-linked network embeddings. Brief Bioinform.

[8] Zhou Y, Amimeur A, Jiang C, Dou D, Jin R, Wang P (2018). Density-aware local Siamese autoencoder network embedding with autoencoder graph clustering.

[9] Zhou Y, Wu S, Jiang C, Zhang Z, Dou D, Jin R, Wang P (2018). Density-adaptive local edge representation learning with generative adversarial network multi-label edge classification.

[10] Yang T, Jiang C, Zhang Z, Dou D, Jin R, Wang P (2019). Integrating local vertex/edge embedding via deep matrix fusion and siamese multi-label classification.

[11] Paulheim H (2016). Knowledge graph refinement: A survey of approaches and evaluation methods. Semant Web.

[12] Pujara J, Miao H, Getoor L, Cohen W (2013). Knowledge graph identification.

[13] Wienand D, Paulheim H (2014). Detecting incorrect numerical data in DBpedia.

[14] Long Y, Wu M, Liu Y, Fang Y, Kwoh CK, Chen J, Luo J, Li X (2022). Pre-training graph neural networks for link prediction in biomedical networks. Bioinformatics.

[15] Yang Q, Yan P, Zhang Y, Yu H, Shi Y, Mou X, Kalra MK, Zhang Y, Sun L, Wang G (2018). Low-dose CT image denoising using a generative adversarial network with Wasserstein distance and perceptual loss. IEEE Trans Med Imaging.

[16] Zhou Z, Wang Y, Guo Y, Qi Y, Yu J (2020). Image quality improvement of hand-held ultrasound devices with a two-stage generative adversarial network. IEEE Trans Biomed Eng.

[17] Choi E, Biswal S, Malin B, Duke J, Stewart WF, Sun J (2017). Generating multi-label discrete patient records using generative adversarial networks.

[18] Li W, Wang Y, Cai Y, Arnold C, Zhao E, Yuan Y Semi-supervised Rare Disease Detection Using Generative Adversarial Network. ArXiv.

[19] Rendsburg L, Heidrich H, Von Luxburg U (2020). NetGAN without GAN: from random walks to low-rank approximations.

[20] Bojchevski A, Shchur O, Zügner D, Günnemann S (2018). NetGAN: generating graphs via random walks.

[21] McCallum AK, Nigam K, Rennie J, Seymore K (2000). Automating the construction of internet portals with machine learning. Information Retrieval.

[22] Wang Y, Yao Q, Kwok JT, Ni LM (2021). Generalizing from a few examples. ACM Comput Surv.

[23] Hochreiter S, Schmidhuber J (1997). Long short-term memory. Neural Comput.

[24] Arjovsky M, Chintala S, Bottou L (2017). Wasserstein generative adversarial networks.

[25] Grover A, Leskovec J (2016). node2vec: Scalable feature learning for networks.

[26] Chen Q, Allot A, Lu Z (2021). LitCovid: an open database of COVID-19 literature. Nucleic Acids Res.

[27] Wei CH, Kao HY, Lu Z (2013). PubTator: a web-based text mining tool for assisting biocuration. Nucleic Acids Res.

[28] Implementation of the paper 'NetGAN: Generating Graphs via Random Walks'. GitHub.

[29] (2020). Repository of the ICML 2020 paper NetGAN without GAN: From Random Walks to Low-Rank Approximations. GitHub.

[30] scikit-learn: machine learning in Python. GitHub.

[31] Shannon P, Markiel A, Ozier O, Baliga NS, Wang JT, Ramage D, Amin Nada, Schwikowski Benno, Ideker Trey (2003). Cytoscape: a software environment for integrated models of biomolecular interaction networks. Genome Res.

[32] Deep denoising of raw biomedical knowledge graph from COVID-19 literature, LitCovid and Pubtator. GitHub.

[33] Dong HW, Yang YH Training generative adversarial networks with binary neurons by end-to-end backpropagation. ArXiv.

[34] Jin W, Barzilay R, Jaakkola T (2018). Junction tree variational autoencoder for molecular graph generation.

[35] Rossanez A, Dos Reis JC, da Silva Torres R, de Ribaupierre H (2020). KGen: a knowledge graph generator from biomedical scientific literature. BMC Med Inform Decis Mak.

[36] Algan G, Ulusoy I (2021). Image classification with deep learning in the presence of noisy labels: A survey. Knowledge-Based Systems.

