# Supplementary material for: Deep Denoising of Raw Biomedical Knowledge Graph From COVID-19 Literature, LitCovid, and Pubtator: Framework Development and Validation
Source: J Med Internet Res. 2022 Jul 6;24(7):e38584. doi: 10.2196/38584 (PMC9301549; doi:10.2196/38584)
Supplement: Multimedia Appendix 1 [file jmir_v24i7e38584_app1.docx]

## **Supplementary 1: Synthetic dataset generation**

First, a positive association graph was constructed from the origin adjacency matrix labeled as 1. Second, a negative association graph was sampled based on the 0 elements in the origin adjacency matrix. Finally, we merged the two types of associations in one graph as our synthetic dataset. (Also, as a restriction of NetGAN, we also colored the minimum spanning tree as red which will be kept in the training set.) An example is shown in (Supplementary Figure 1).


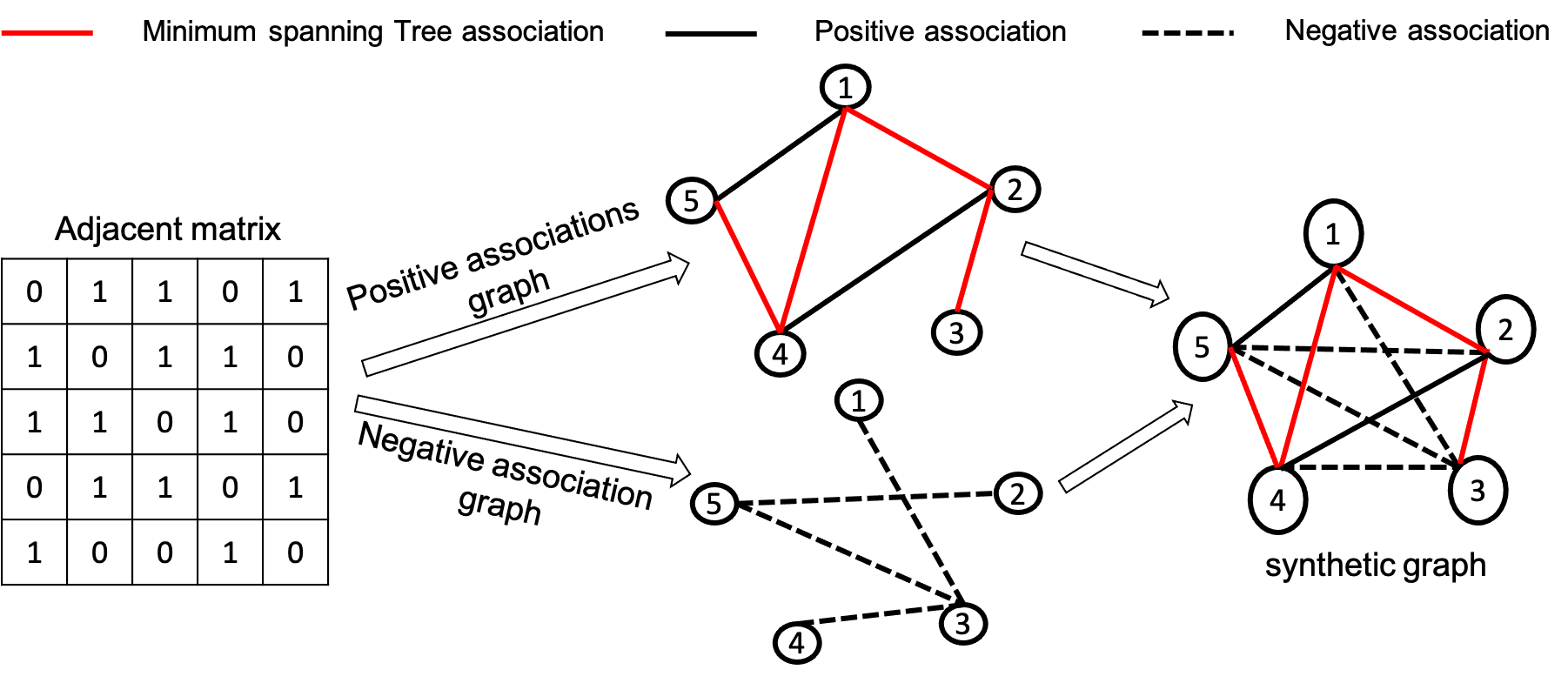


Figure S1: Synthetic dataset construction pipeline

## **Supplementary 2: Real dataset preprocessing and statistic**

(Supplementary Table 1) shows the detailed number of associations we preprocessed and collected from the raw data, as well as data filtered from the Gene-Chem associations (by requiring that the vertices must appear in our previous two types of associations) (Chem-Dise, Gene-Dise). After merging the identical node id within the same category, we got a total of 23587 nodes. (Supplementary Table 2) shows the number of the three types of edges and the corresponding labeled ground truth. Except for the histogram of degree in all associations reported in the manuscripts. Here we report the histogram degree of each type of association in our (Supplementary Figure 2).

Table S1. The raw dataset with node numbers counted from Litcovid and Pubtator.

|  | **Litcovid** | **Pubtator** |
| --- | --- | --- |
| Chem-Dise | 1572-104 | 3728-7748 |
| Gene-Dise | 2304-124 | 5146-8540 |
| Gene-Chem(filtered) | 1499-1318 | 4725-3352 |
| Gene-Chem(queried) | 2350-2014 | 20881-13518 |

Table S2. Constitution of our real dataset with different types of edges

| **type** | **Number** | **Labeled (ground truth)** |
| --- | --- | --- |
| Gene-Chem | 228148 | 350 |
| Gene-Dise | 32611 | 100 |
| Chem-Dise | 27511 | 50 |
| total | 288270 | 500 |

| 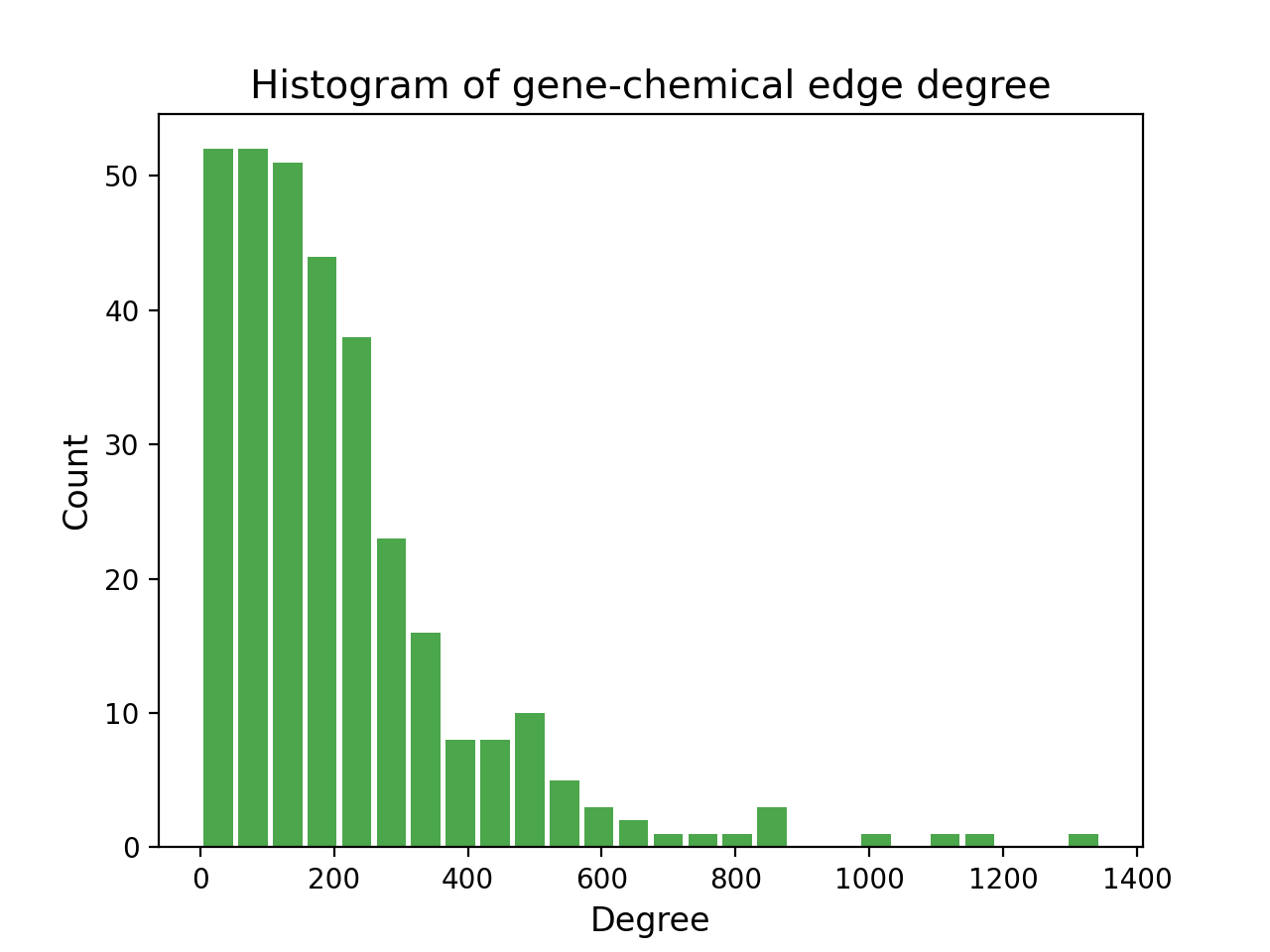 |
| --- |
| (A) Gene-chemical |
| 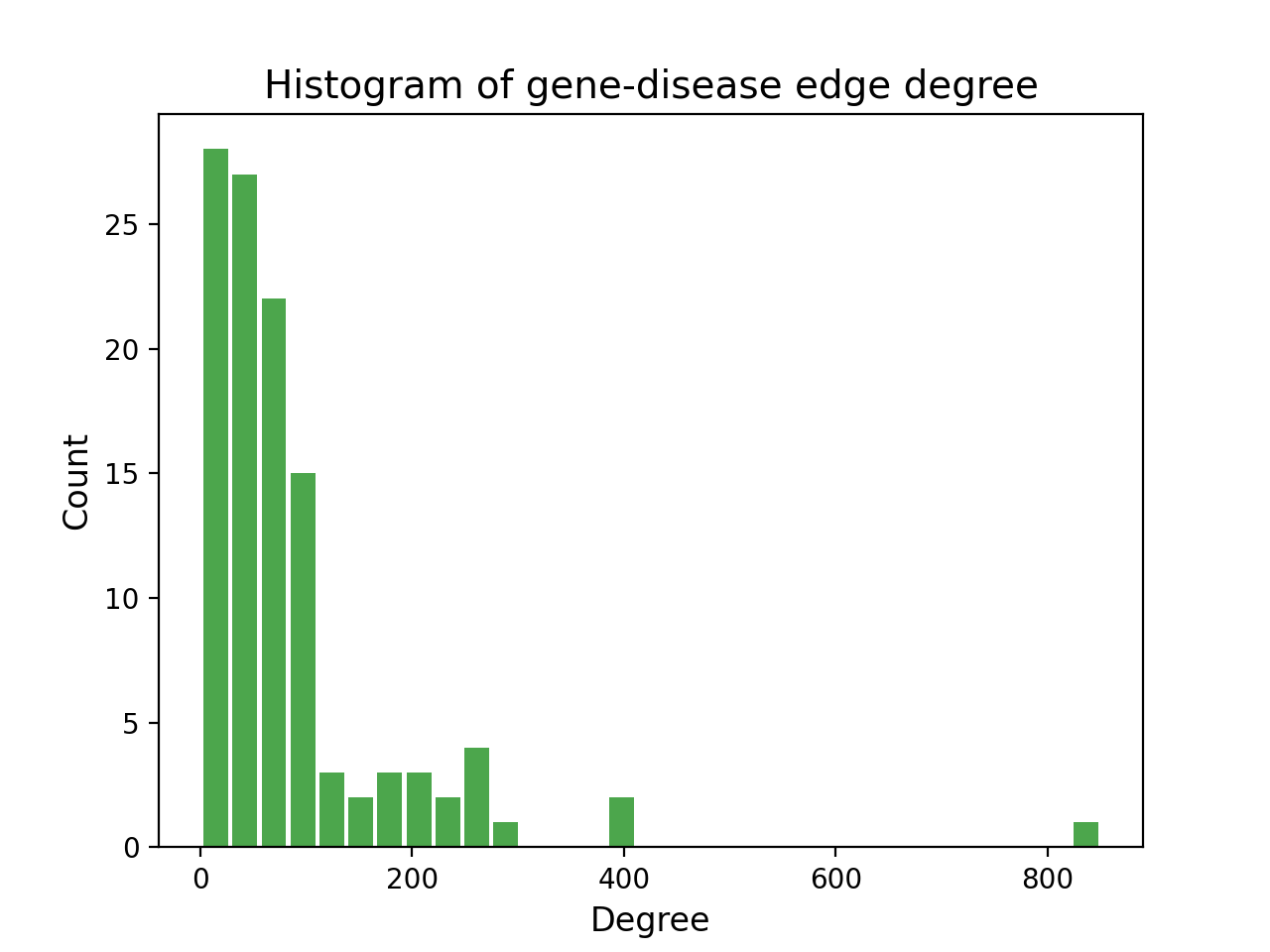 |
| (B)Gene-disease |
| 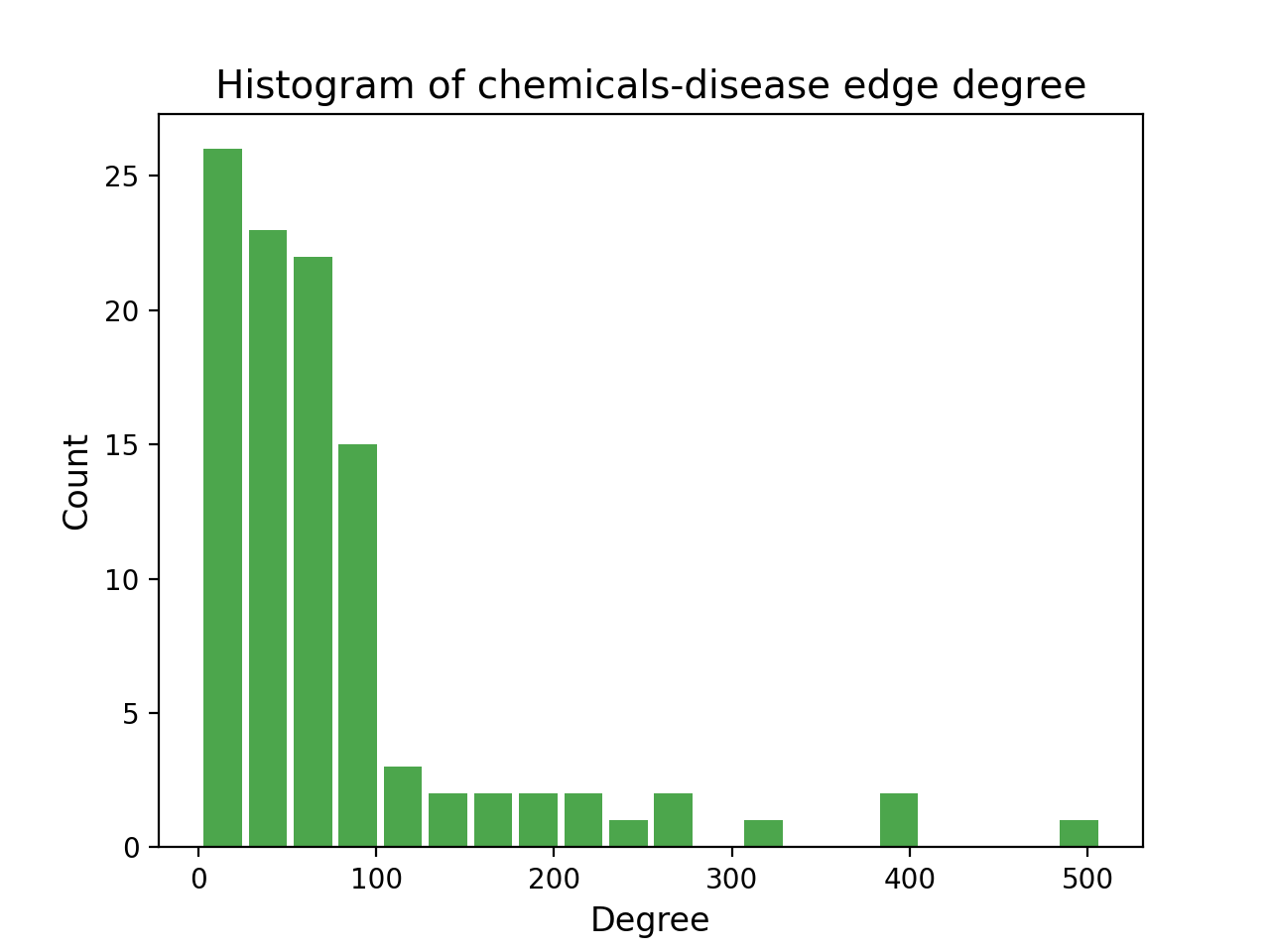 |
| (B) Chemical-disease |

Figure S2. Degree of three types of edges in real dataset

## **Supplementary 3: Average Precision report in synthetic and real datasets**


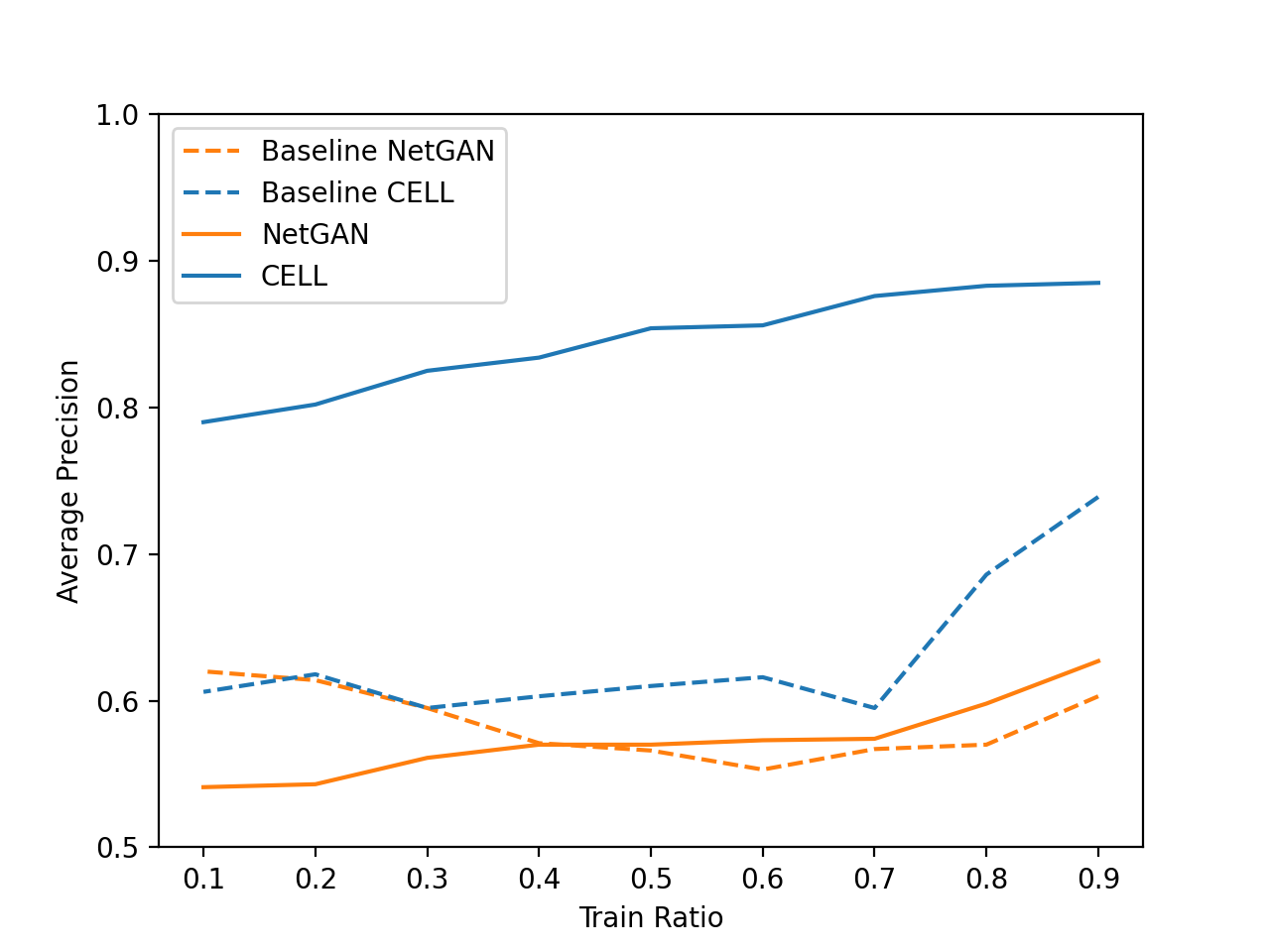


Figure S3. Average Precision performance of NetGAN and CELL with/without unlabeled information in synthetic dataset


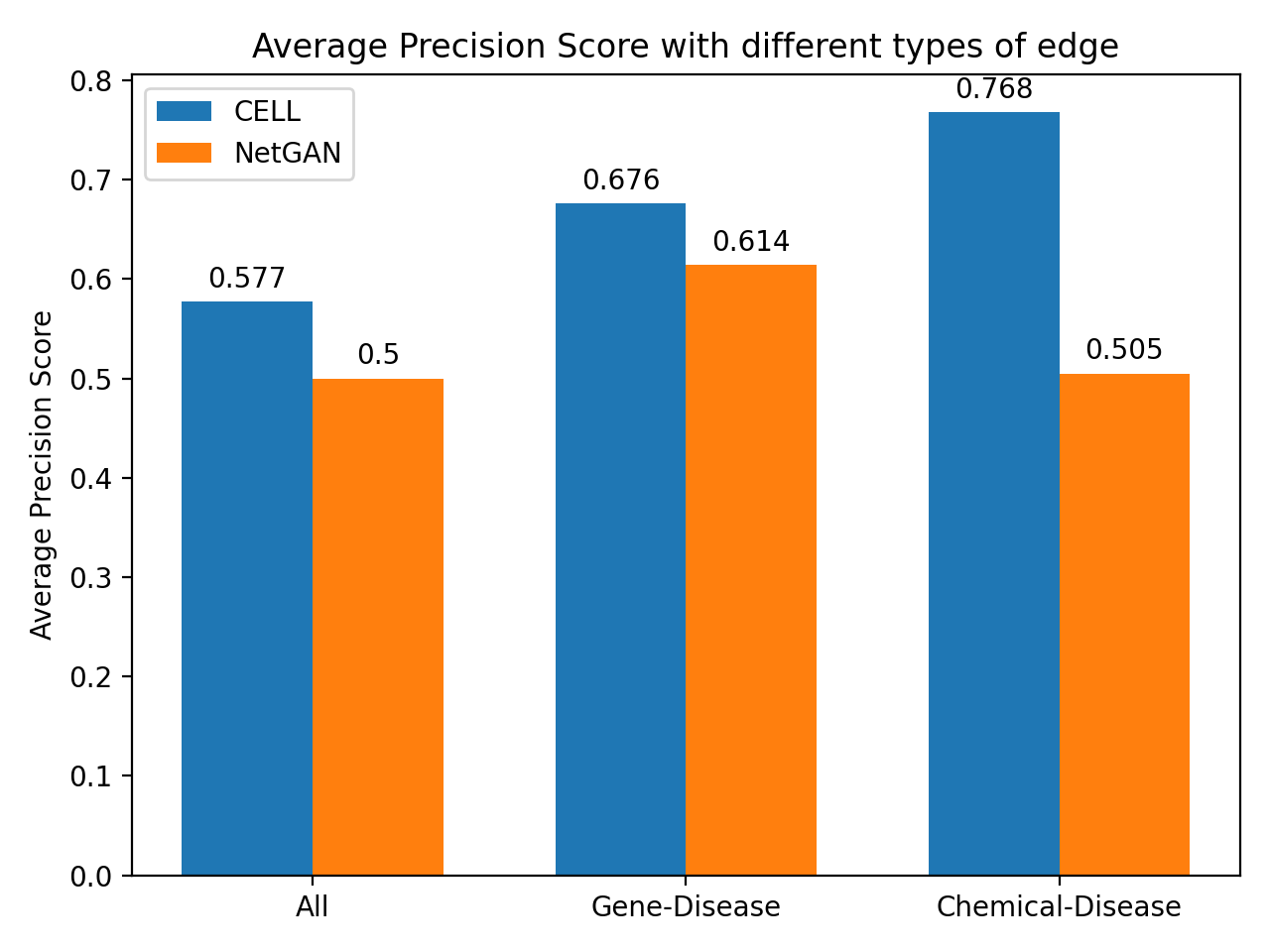


Figure S4. Average Precision Score for different types of associations in real dataset.
